# Brucellosis: An Elusive Backyard Agent

**DOI:** 10.7759/cureus.8154

**Published:** 2020-05-16

**Authors:** Zain Rizvi, Tahir Iqbal, Haider Bokhary, Shiza Chaudhry

**Affiliations:** 1 Internal Medicine, Shifa International Hospital, Islamabad, PAK; 2 Medicine, Shifa International Hospital, Islamabad, PAK

**Keywords:** brucella, brucellosis, infectious disease, medicine, internal medicine, family medicine, contagious, zoonotic, zoonosis, pasteurization

## Abstract

Brucellosis is a form of zoonotic infection caused by various *Brucella *organisms. It most commonly presents as a case of pyrexia of unknown origin, alongside symptoms such as night sweats, malaise, arthralgias, and myalgias. This report describes the case of a man who presented with pyrexia of unknown origin for one month; he was diagnosed to be a case of brucellosis after enteric fever was ruled out. Investigations were ordered as it was a differential diagnosis with high clinical suspicion due to the presenting complaint and potential exposure of tainted consumable products. The systemic disease was determined to be brucellosis following blood results demonstrating positive antibody titers, and the suspicion of exposure due to widespread inadequacies in sterilization of food products.

## Introduction

Brucellosis, also known as Mediterranean fever, is a zoonotic infection caused by animals infected with *Brucella *organisms, which are aerobic intracellular coccobacilli. Humans are accidental hosts; it is transmitted to them by means of infected animal fluids or tissue, or by ingestion of animal food products that have not been sterilized or pasteurized [1]. It presents with insidious onset of fever, night sweats, malaise, and arthralgias. Uncommonly, it can cause weight loss, cough, and abdominal pain [2].

The burden of the disease remains high, approximately 500,000 cases are reported worldwide every year, the majority being in the Middle East, Asia, the Indian Subcontinent, and South America [3,4]. The most common cause of infection is due to ingestion of food products that are not sterilized or pasteurized properly [1]. Furthermore, brucellosis caused due to infection from wildlife is another possible mode of transmission to humans or domestic animals that may pass it on to their handlers, which is a form of occupational risk associated with the disease [5]. Four organisms can cause infections in humans, each one being from a different infected species of animals. *Brucella melitensis* from sheep and goats, *Brucella abortus* from cattle, *Brucella suis* from swine, and *Brucella canis* isolated from dogs. From the listed species, the most virulent infection is due to sheep, goats, and swine [6].

Brucellosis typically presents with a fever in around 80% of patients; this leads to the diagnosis of fever of unknown origin in some areas where it is rare [7]. Secondly, chills are very common in these patients. Constitutional symptoms include weight loss, malaise, fatigue, and arthralgias, which are present in around 55% of presenting cases [8]. The nervous system is rarely involved but can lead to neuropsychiatric symptoms, such as headache, weakness, and depression. Furthermore, gastrointestinal symptoms typically range from dyspepsia to abdominal pain caused by hepatic abscesses [1,8]. Rarely there can be cough and dyspnea, these are typically due to an underlying empyema [9]. Lastly, in its chronic form which lasts for longer than one year, there is an afebrile pattern, alongside history of myalgia, fatigue and arthralgias. This frequently leads to a differential diagnosis of chronic fatigue syndrome [8,10].

Hence, as brucellosis can mimic numerous diseases due to its varied symptoms, it is important to look for clues such as a person’s occupation, the area that they live in, and know about the endemicity of *Brucella *in that region. Due to the organism’s predominance in the region of the presenting patient, it was considered as a differential diagnosis of this patient’s condition.

We illustrate a case of an old man who presented with a step ladder pattern fever, myalgias, dry cough, fatigue, and reduced appetite. Investigations showed a positive blood test for brucella immunoglobulin M (IgM) antibodies, alongside findings on x-ray and computed tomography (CT) scan.

## Case presentation

A 58-year-old retired man of Pakistani origin, known smoker, presented to our outpatient clinic with fever for the past one month. It was associated with painful body aches, dry cough, reduced appetite, and fatigue. The fever had been present for every day, and it was of a step ladder pattern, with a rise during the evening time. It achieved a maximum of 102°F in the evening and it reduced to approximately 99°F during the early morning with associated chills and rigors. The patient did not report any other symptoms. There was no significant past medical history. He was prescribed oral amoxicillin by a local doctor previously for his symptoms with no improvement noted.

On physical examination, an ill looking man with a body temperature of 101°F, a pulse rate of 76/min, a blood pressure of 120/70 mmHg, and a respiratory rate of 18 breaths/min. On inspection, there was no apparent rash, marks, or swellings present. On palpation, no tenderness was present. Respiratory and abdominal system examinations were normal and had no positive findings. There was no lymphadenopathy or hepatosplenomegaly.

Investigations revealed normal hemoglobin (14.7 g/dL) and platelet count (269 × 10^3^/mm^3^). However, white blood cell (WBC) counts were raised (10,800/mm^3^) and differential counts were skewed (neutrophils 66%, lymphocytes 20%, monocytes 9%, and eosinophils 5%). Further blood biochemistry showed normal blood sugar fasting (108 mg/dL) and thyroid function tests; he had low levels of folic acid (2.3 ng/mL) and 25-hydroxy vitamin D (11.39 ng/mL). Liver function tests revealed mildly raised serum glutamic pyruvic transaminase (SGPT) (43 IU/L) and increased levels of gamma-glutamyl transferase (GGT) (94 U/L). C-reactive protein (CRP) and erythrocyte sedimentation rate (ESR) were measured and were increased (71.62 mg/L and 49 mm/first hour). Malarial infection was excluded out by means of a negative blood smear test. Sputum smear for acid fast bacilli revealed no growth. However, blood test for brucella IgM antibodies was positive (1.31). Initial chest radiograph did not show any anomaly.

Due to the prolonged pyrexia of unknown origin initially, a preliminary diagnosis of enteric fever was made. He was admitted to the hospital in-patient department under the care of a specialist in internal medicine. The initial diagnosis was suspected to be enteric fever, based on the endemicity in the area, and accordingly he was started on azithromycin and ceftriaxone therapy. Blood tests done later during the admission revealed the presence of brucella antibodies, leading to the definitive diagnosis of brucellosis and change in treatment plan.

**Figure 1:**
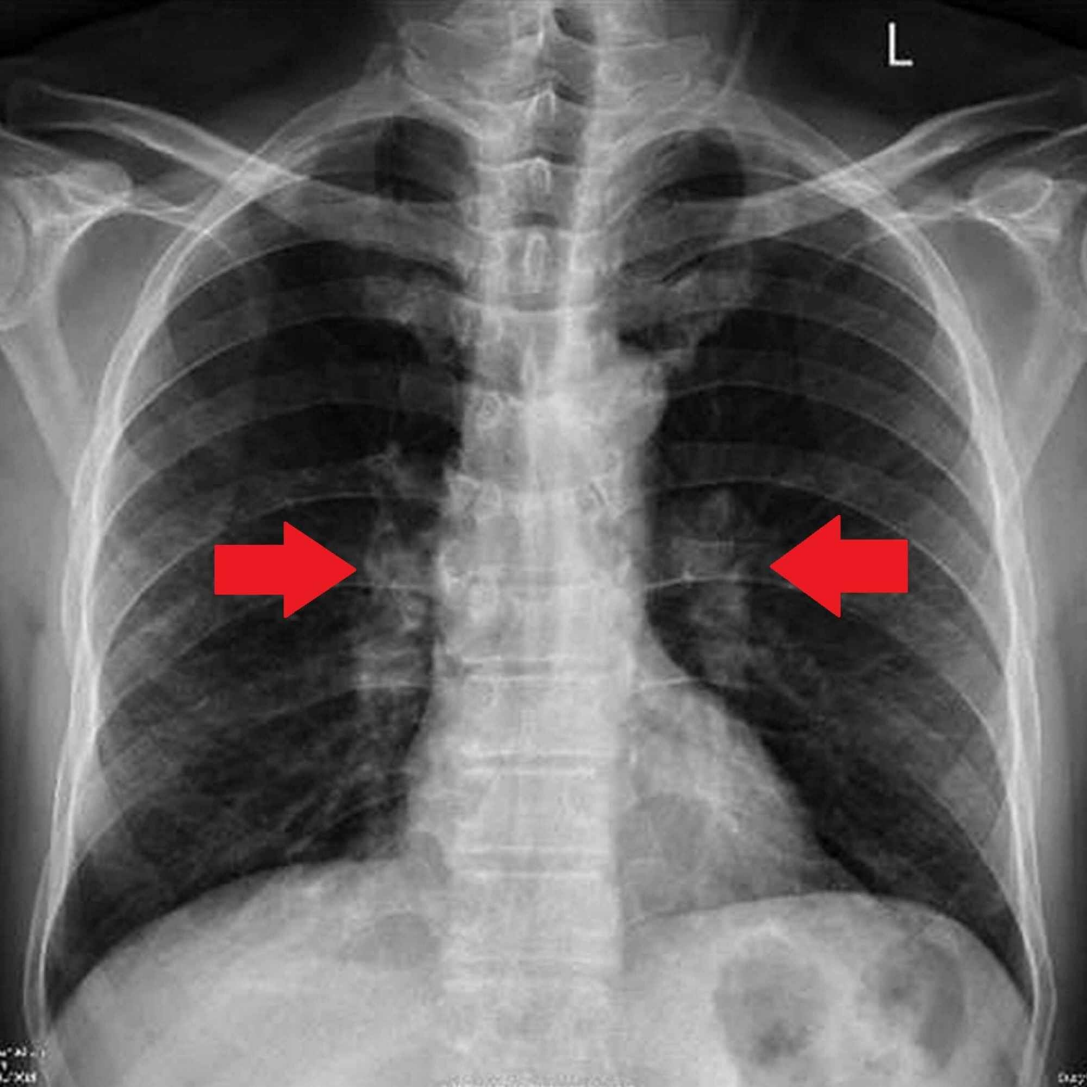
Chest x-ray before treatment with doxycycline with presence of bilateral hilar lymphadenopathy (red arrows).

Furthermore, imaging studies three days after admission consisted of a chest radiograph, which showed mildly prominent subcentimeteric mediastinal hilar lymph nodes and emphysematous changes in lungs (Figure 1). CT of the chest was ordered, and it showed multiple subcentemetric mediastinal hilar lymphadenopathy. The diagnosis of brucellosis was made due to the presence of brucella antibodies in the blood report. Additionally, this seemed like an appropriate diagnosis due to *Brucella* being silently prevalent in the rural areas of Pakistan. Human brucellosis is known to cause a wide array of symptoms, of which some were present in the presenting case. Following confirmation of brucellosis, he was treated with doxycycline (100 mg twice daily) and rifampicin (600 mg once daily). After treatment was completed, the patient was discharged with a three-week follow-up to the medical clinic.

## Discussion

Brucellosis is a condition that puts enormous burdens in the countries where it is endemic. The fact that it is endemic to countries mostly lacking adequate resources and infrastructure to diagnose all cases leads to a high possibility that most cases of brucellosis remain undetected under the guise of pyrexia of unknown origin. This is likely one of the reasons the World Health Organization (WHO) has categorized it as a “neglected zoonotic disease” [11]. With early detection and the right treatment, the prognosis of brucellosis is generally excellent. However, if left untreated, it can lead to several complications ranging from neurobrucellosis to endocarditis, conditions which significantly increase the threat of morbidity and mortality of the disease [12]. Therefore, recognizing the signs and symptoms of brucellosis, and subsequently diagnosing and treating the patient are of paramount importance.

Fever is the most common symptom of an acute infection with *Brucella*, while in chronic cases the most common symptom is arthralgias [13]. The fever can develop either suddenly or insidiously and more commonly follows an undulant pattern. Several other symptoms can develop along with fever and arthralgias as was the case in the patients being reported, with other symptoms including cough, loss of appetite, and fatigue. Another rare symptom is testicular pain and swelling in patients with active brucellosis, with one study reporting an incidence of 10% [13]. There are several cases where enlargement of liver, spleen, and lymph nodes has been observed along with the fever, as a result of which it becomes important to rule out lymphomas as well [14]. This was also seen in our patient with the development of hilar lymphadenopathy on follow-up chest radiography.

Hematological parameters are frequently disturbed in brucellosis. While our patient’s blood picture revealed leukocytosis (10,800/mm^3^) with a normal red cell and platelet count, the more common findings in brucellosis include leukopenia and relative lymphocytosis along with anemia, thrombocytopenia, and rarely pancytopenia [15]. Hypersplenism, which was absent in our patient, appears to play a fundamental role in producing these abnormalities of the peripheral blood [15]. Like in any other infection, elevation in inflammatory markers like ESR and CRP is expected to be seen. In certain studies, 38% to 87% and 34% to 81% of patients with brucellosis had elevated ESR and CRP, respectively [16]. As expected, our patient showed increased levels of both inflammatory markers. Similarly, a slight elevation in liver enzyme levels is a very common finding, as was seen with a slight increase in SGPT and GGT in the patient. These elevated levels may reflect the severity of hepatic involvement and correlate clinically with hepatomegaly.

Multiple organ systems can be affected due to brucellosis, resulting in a variety of clinical presentations. Our patient reported experiencing dry cough; subsequent chest radiography and CT showed subcentimeteric mediastinal hilar lymphadenopathy. Although pulmonary involvement is rare in brucellosis, the following radiological abnormalities have been reported: miliary mottling, parenchymal nodules, pleurisy, lung abscess, bronchiectasis, pneumonic consolidation, hilar or paratracheal lymphadenopathy, and pneumothorax [17].

The standard treatment of choice for brucellosis according to literature comprises a doxycycline-aminoglycoside combination as the first choice, followed by doxycycline-rifampin and doxycycline-cotrimoxazole as alternatives. For children younger than eight years of age, the antibiotic combination used is cotrimoxazole and rifampin [18]. Our patient received a combination of doxycycline and rifampicin, which led to recovery.

It is important to note that our patient was not affiliated with an occupation predisposed toward acquiring the infection. Therefore, it becomes vital to gather data regarding diet, travel and occupation from patients suspected of brucellosis. It has been observed that the most common risk factors for development of the infection are consumption of unpasteurized milk (48%) and contact with livestock animals (59%) [19]. Occupations predisposed toward such exposure most commonly include farmers, livestock breeders, and shepherds, but are also common among veterinarians and laboratory workers [20].

## Conclusions

Brucellosis is a condition considered by the WHO to be “neglected zoonotic disease,” which, although has good prognosis with the right treatment, can become much more complicated if left undetected and untreated. It is also rare enough a condition to be missed among the many causes of fever of unknown origin especially in areas with lower endemicity. What aided us in the diagnosis in our patient were the relatively typical presenting symptoms having lasted for one month, the infrequent pasteurization of milk in our country, and the access to adequate diagnostic facilities in our set up to detect a rise in WBC counts, ESR, CRP, and the eventual detection of brucella IgM antibodies, basic facilities which are still not readily accessible to many people of our country. This study highlights how it may be an important cause of fever even in patients naïve to the typical predisposing factors and in areas of the world not thought to be particularly endemic.
